# The Role of Gender in the Association Among the Emotional Intelligence, Anxiety and Depression

**DOI:** 10.3389/fpsyg.2021.747702

**Published:** 2021-10-04

**Authors:** Maria Rita Sergi, Laura Picconi, Marco Tommasi, Aristide Saggino, Sjoerd J. H. Ebisch, Andrea Spoto

**Affiliations:** ^1^Department of Medicine and Aging Sciences, University of G.’ d’Annunzio, Chieti, Italy; ^2^Department of Neurosciences, Imaging and Clinical Sciences, Chieti, Italy; ^3^Department of General Psychology, University of Padua, Padua, Italy

**Keywords:** emotional intelligence (E.I.), depression, anxiety, nomological association, gender differences

## Abstract

Recent epidemiological data show an increase of depression and anxiety that cause a loss of about 3–4% of the gross domestic product in Europe, as a consequence of a reduced productivity and a premature death of people. Gender differences in both psychopathologies were found from mid-to-late adolescence until 55 years, and data indicate an increase of depression in women. Considering these data, new interventions focused on promoting psychological well-being were designed. A predictive factor of psychological disorders is Emotional Intelligence (EI), the ability to understand and regulate our own emotions, as well as those of others. EI is associated with psychological well-being, as well as with the treatment of mental illness, but gender differences in the association among EI, anxiety and depression remains unclear. The present study aims at analyzing the nomological associations among EI, anxiety and depression. Furthermore, the possible moderating role of gender in the relation between EI, depression and anxiety is investigated in a sample of 1725 healthy participants. Our results show that the ability to recognize and to control emotions in the social context helps us to reduce the risk to be affected by depression and anxiety. Moreover, our study shows that the association of EI with anxiety and depression wasn’t gender moderated. In conclusion, the findings highlight that EI can help people to manage emotions linked to negative events and to successfully understand emotions in others. In addition, we found no moderation role of gender in the association between EI, anxiety and depression.

## Introduction

Recent epidemiological data underline an increase of mental disorders (World Health Organization; World Health Organization [WHO], 2020a, b) according to two indicators: Years Lived with Disability (YLDs) and Disability-Adjusted Life Years or DALYs. Years Lived with Disability is a measure of years lived with a disease; DALYs is a measure of numbers of years lost due to diseases. Between 2007 and 2017, depression was the third leading cause of YLDs with an increase of 14.3% (Global Borden of Disease [GBD], 2018). In addition, anxiety and depressive disorder were among the highest causes of DALYs in 2019 (Global Borden of Disease [GBD], 2020). Depression can lead to suicide, which is the second leading cause of death in people with ages between 15 and 29. Depression and anxiety are among the most common mental disorders with a relevant socioeconomic impact. Recent data show that mental disorders cause a loss of about 3–4% of the gross domestic product in Europe, as a consequence of a reduced productivity and a premature death of people suffering from psychopathologies (De Girolamo et al., 2005; World Health Organization [WHO], 2016; Italian National Institute of Health [ISS], 2020a). In Italy, in the years between 2016 and 2019, 6% of the adult population reported depressive symptoms and having a deteriorated well-being (Italian National Institute of Health [ISS], 2021). Considering these data, new interventions were designed that focused on promoting psychological well-being and on preventing the onset of mental disorders through effective psychotherapeutic and pharmacological treatments (Kendrick and Pilling, 2012; World Health Organization [WHO], 2017, 2020c; Italian National Institute of Health [ISS], 2020b).

Depression and anxiety are the common causes of DALYs among women in 2019. Gender differences in Major Depressive Symptoms (MMD) were found from mid-to-late adolescence until 55 years, and data indicate an increase of depression in women (Global Borden of Disease [GBD], 2020). No significant gender differences between 55 and 65 were found (Girgus and Yang, 2015). Indeed, stressful life events and biological factors explain gender differences: women are more exposed to trauma than men; moreover, ovarian hormones are linked to mood changes. In particular, women process traumatic events worse than men due to a hypothalamic-pituitary-adrenal (HPA) axis’s dysregulation. This dysregulation leads to an elevated cortisol response to stress. In addition, ovarian hormones modulate the HPA axis. The HPA axis’s dysregulation increases during periods of change of ovarian hormones (e.g., postpartum period). This aspect leads to a difficulty to regulate the stressor, making women more vulnerable to depressive symptoms (Nolen-Hoeksema et al., 1999; Weiss et al., 1999; Nolen-Hoeksema, 2001; Oh et al., 2018). Gender differences in anxiety occur in middle age and they decline after the age of 65, due to neurobiological factors (Jalnapurkar et al., 2018). Hypothalamic-pituitary-adrenal (HPA) axis’s dysregulation contributes to mood regulation (Altemus et al., 2014).

An important predictive factor of psychological disorders that needs further exploration is Emotional Intelligence (EI), that is, the ability to understand and regulate our own emotions, as well as those of others (Salovey and Mayer, 1990). Mayer and Salovey (1997) defined EI as a form of “intelligence” composed by four cognitive abilities: the evaluation and the expression of emotions; the regulation of emotions; the use of emotions for solving problems; the emotive regulation. Petrides and Furnham (2000, 2001, 2003) introduced two new concepts related to EI: “Trait EI” or “Trait Emotional Self-Efficacy” and “Ability EI.” The first concept indicates a series of self-perceptions concerning the ability to identify emotions; the second concept concerns a “cognitive-emotional ability.” “Trait EI” is measured via self-reporting instruments (e.g., Emotional Intelligence Scale; EIS; Schutte et al., 1998; Emotional Quotient Inventory; EQ-i; Bar-On, 1997; Trait Emotional Intelligence Questionnaire; TEIque; Petrides and Furnham, 2009); “Ability EI” is measured through maximum performance tests (e.g., Mayer – Salovey – Caruso Emotional Intelligence Test; MSCEIT; Mayer et al., 2002; Petrides et al., 2016).

EI is associated with psychological well-being and with the treatment of mental illness (Brackett et al., 2004; Austin et al., 2005; Martins et al., 2010; Di Fabio, 2011; Schutte and Malouff, 2011; Picconi et al., 2019). In particular, Trait EI is negatively associated with anxiety and depression. Suicidal thoughts related to depression disorder was found to be decreased in individuals with a high ability to understand emotions and a good self-control (Bauld and Brown, 2009; Armstrong et al., 2011). Depressed persons have a lower ability to understand, to express emotions and to manage negative emotions (Fernández-Berrocal et al., 2006; Sergi et al., 2012). In the clinical context it’s of great clinical relevance that the ability to recognize and to control emotions in social contexts reduces the risk for depression and anxiety. Indeed, the inability to control negative emotions is associated to stress and depression, because there is a difficulty of emotional expression (Batool and Khalid, 2009). In particular, a recent review demonstrated an association between self-report EI tests and suicide risk in people with major depressive disorders (Domínguez-García and Fernández-Berrocal, 2018). For example, high scores in managing self-emotions correlated negatively with suicidal behavior tendency (*r* = −0.41; *p* < 0.001) (Ciarrochi et al., 2002). Regarding anxiety disorders, several studies reported negative correlations between trait EI and anxiety in university students. For example, Jan et al. (2020) reported negative correlations between EIS’s factors of Perception of Emotions, Managing own Emotions, Managing others’ Emotions and Utilization of Emotions and anxiety in a sample of university students (*r* = −0.182; *p* < 0.01; *r* = −0.251; *p* < 0.01; *r* = −0.237; *p* < 0.01; *r* = −0.197; *p* < 0.01, respectively). Ahmadpanah et al. (2016) showed negative correlations between EQ-i’s factors of Intrapersonal skills, Interpersonal skill, Stress Management, Adaptability and General Mood (*r* = −0.65; *p* < 0.001; *r* = −0.61; *p* < 0.01; *r* = −0.55; *p* < 0.01; *r* = −0.56; *p* < 0.01; *r* = −0.61; *p* < 0.001, respectively). Several studies showed that emotional dysregulation is one of the predictors of anxiety and depression (Downey et al., 2008, 2010). Certainly, a proper emotional regulation involves inhibitory processes that suppress the generation of inadequate emotional states and, consequently, a better individual adaptation. Therefore, the ability to use effective regulatory strategies increases psychological well-being, while the inability to regulate emotions leads to a poor mental health. Finally, emotional regulation permits a good control of ruminative thoughts generated by anxiety and depression (Galeazzi and Meazzini, 2004; Fernández-Berrocal et al., 2005; Lim and Kim, 2005).

Gender differences in the association between EI, anxiety, and depression have been studied, but results are inconsistent. Some authors found no significant differences in self-report EI tests between males and females (Petrides and Furnham, 2000; Fernández-Berrocal et al., 2004; Poulou, 2010). Other studies, on the contrary, showed that EI scores were higher in females than in males (Ciarrochi et al., 2001; Schutte et al., 2002; Katyal and Awasthi, 2005; Van Rooy et al., 2005a; Craig et al., 2009; Whitman et al., 2009). To our knowledge, only one study investigated the moderation effect of gender on the relationship between EI and depression, indicating a negative relation between ability EI and depressive disorders in men (β = −0.12; *p* < 0.01) (Salguero et al., 2012). No studies analyzed the effect of gender on the relationship between perceived EI, anxiety and depression. Despite the connection between EI and anxiety and depression, many critiques arose concerning the scientific validity of the EI construct. The major criticism concerns the existence of different and, in some cases, contradictory models of EI (Bechara et al., 2000; Brackett and Mayer, 2003; Van Rooy and Viswesvaran, 2004; Huang et al., 2006; Cherniss, 2010). Many authors argue that studies need to be increased to better define the number and structure of EI dimensions (Davies et al., 1998; Ciarrochi et al., 2000; Newsome et al., 2000; Van Rooy et al., 2005b; Di Fabio, 2011; Hansenne, 2011; Rullo et al., 2015). As a consequence of the presence of competing models of EI, some authors underline the urgency to redefine the dimensions and the terminology of EI through the development of valid and reliable assessment instruments (Tapia and Marsh, 2006; Hussein et al., 2019). In psychological research, there are two types of psychometric instruments for the measurement of EI: self-report and ability tests (Saggino et al., 2013). The principal debate is about the method of *scoring* in ability tests, due to a difficulty to use an objective method to score the experience of emotions (Zeidner et al., 2012), whereas the issue of self-report instruments of EI is the unclear factorial validity. Self-report instruments measure perceived EI (PEI) or self-perceptions (Van Rooy and Viswesvaran, 2004; Keele and Bell, 2008; Gong and Paulson, 2018). Because of its brevity and large availability, the Emotional Intelligence Scale (EIS; Schutte et al., 1998) is among the most used self-report instruments to evaluate PEI. Emotional intelligence scale consists of 33-items with 5-Likert response scale (from 1 = “Totally disagree” to 5 = “Totally agree”) and it is based on Salovey e Mayer’s model of EI. The major limit of EIS is related to the ambiguity of its factorial structure (Tapia and Marsh, 2006; Zhoc et al., 2017; Gong and Paulson, 2018). Di Fabio et al. (2008) analyzed the EIS dimensional structure in a sample of Italian adults. A three-factors solution has been found: “Evaluation and Expressions of Emotions” (α = 0.84), “Regulation of Emotions” (α = 0.82) and “Use of Emotions in Problem Solving” (α = 0.79). Grazzani Gavazzi et al. (2009) analyzed EIS factor structure in an Italian adolescents’ sample. Also in this case, the authors found a three-dimensions structure: “Evaluation of Emotions to Others” (α = 0.73), “Evaluation of Emotions to Self” (α = 0.66) and “Regulation of Emotions” (α = 0.72). Ciucci et al. (2009) tested this factorial structure in a sample of Italian pre-adolescents, obtaining reliable measure for each factor: α = 0.68 for “Evaluation of Emotions to Others”; α = 0.64 for “Evaluation of Emotions to Self”; α = 0.71 for “Regulation and Use of Emotions.” However, other scientific studies did not find a consistent factor structure of the EIS, both in samples composed by university students (Petrides and Furnham, 2000; Ciarrochi et al., 2002; Saklofske et al., 2003; Austin et al., 2004; Jonker and Vosloo, 2008; Ng et al., 2010; Zhoc et al., 2017; Gong and Paulson, 2018; Hussein et al., 2019; Adamakis and Dania, 2021) and by adults (Austin et al., 2005; Gignac et al., 2005). Finally, very few studies have examined the generalizability of the EIS’ structure in males and females (Grazzani Gavazzi et al., 2009). There is a little knowledge about structural and measurement invariance between sex in trait EI (Tsaousis and Kazi, 2013).

Considering these data, the first aim of this study was to analyze the factor structure of EIS and its validity and reliability. The second aim was to analyze EIS measurement invariance between genders. The third aim was to study the nomological associations between EIS, anxiety, and depression scores. Finally, the possible moderating role of gender in the relation between EI, depressive and anxiety score was investigated. On the basis of our aims and previous literature we hypothesize a model with four-factor dimensions of trait EI (Petrides and Furnham, 2001; Saklofske et al., 2003; Hussein et al., 2019); we hypothesize the same factor structure between gender of the tested model (Grazzani Gavazzi et al., 2009); we hypothesize that high EI scores are associated to lower levels of depression and anxiety (Fernández-Berrocal et al., 2006; Sergi et al., 2012); finally, no we hypothesize that no moderating role of gender on the relationship between trait EI, anxiety and depression.

## Materials and Methods

### Participants and Procedure

1725 participants, (62.2% females) were included in the study on a voluntary basis. The participants were representative sample of the normal population. The mean age of the total sample was 25.68 years (SD = 11.34); the mean age for female’s sample was 24.75 (DS = 10.479); the mean age for male’s sample was 27.19 (DS = 12.489). Eighteen participants (1.0%) did not declare their age. All procedures performed in studies involving human participants were in accordance with the ethical standards of the institutional and national research committee and with the 1964 Helsinki declaration and its later amendments or comparable ethical standards. Anonymity and privacy of the participants were guaranteed according the Italian and the European laws about privacy (Italian law n. 196/2003 and EU GDPR 679/2016, respectively). Informed consent was obtained from all individual participants included in the study. Participation was voluntary. Furthermore, the sample was heterogeneous in terms of the age range (maximum 70 years old). Indeed, the sample was composed by university students and workers. The most of workers were housewives, liberal professions and teachers. The questionnaires were administered by a trained person in psychometrics methodology and clinical practice. Participants signed an informed consent in which the respect of the privacy of their data (even if questionnaires were in anonymous format) was declared and the main aim of the research was explained. The study was approved by the Department of Medicine and Aging Sciences, University of Chieti, Italy.

### Measures

#### Emotional Intelligence Scale

The EIS (Schutte et al., 1998) is a self-report scale with 33-items. Scores are on a five levels Likert scale (from 1 = “Totally disagree” to 5 = “Totally agree”).

#### Teate Depression Inventory

The Teate Depression Inventory (TDI) (Balsamo and Saggino, 2013, 2014; Balsamo et al., 2014) consists of 21 items which measures major depressive disorders, according to the latest edition of the Diagnostic and Statistical Manual of Mental Disorders (DSM-5; American Psychiatric Association [APA], 2013). The severity of each symptom is rated on a five levels Likert scale ranging from 1 (“Never”) to 5 (“Always”).

#### Beck Anxiety Inventory

The Beck Anxiety Inventory (BAI; Beck and Steer, 1993) is a self-report scale for screening anxiety disorders. Item scale was a 4 levels Likert scale ranging from 0 (“Not at all”) to 3 (“Severely”).

### Statistical Analyses

Missing values have been replaced by the variable mean (Pigott, 2001). Means and standard deviations were computed, and skewness and kurtosis were estimated to analyze data distributions (Ercolani and Perugini, 1997). The internal consistency of the psychological scales was estimated with McDonald’s omega (Zinbarg, 2005; Dunn et al., 2014).

The sample was divided randomly into two sub-samples (Bollen, 1986). The factorial validity of the EIS was analyzed via *Explorative Factor Analysis* (EFA) in the first sub-sample (*n* = 828). The *maximum Likelihood* extraction method was used; the criteria used for determining the number of factors were the *scree plot* (Cattell, 1966) and the eigenvalue > 1 (Kaiser, 1974). The Oblimin rotation was used for oblique factor rotation. Items with factor loadings less than |0.30| and/or with multiple saturations were removed. *Confirmatory Factor Analysis* (CFA) was conducted on the second sub-sample (*n* = 897) to confirm the factor structure of the first analysis (Floyd and Widaman, 1995). The adequacy of the factor model was assessed using Goodness-of-Fit Indices: traditional Chi-Square (χ^2^) goodness of fit test and its degrees of freedom, the Root-Mean-Square Error of Approximation (RMSEA), the Comparative Fit Index (CFI), the Goodness of Fit Index (GFI), the Adjusted Goodness of Fit Index (AGFI). The cutoff values for a satisfactory model were: RMSEA ≤ 0.08; CFI ≤ 0.95; GFI ≤ 0.90; AGFI ≤ 0.85. The acceptable values were: CFI < 0.90 – 0.94 >; RMSEA = 0.08. The cutoff values for a good model were: CFI ≥ 0.95; RMSEA = 0.06 (Hu and Bentler, 1999; Schermelleh-Engel et al., 2003; Steiger, 2007; Savalei and Bentler, 2010; Tarka, 2018). Hu and Bentler (1999) recommended a cutoff of 0.95 for CFI.

To study measurement invariance of the EIS between genders, a Multigroup Confirmatory Factor Analysis (MG-CFA) was performed. The MG-CFA started with separate baseline CFA models that were tested separately for males and females. In the configural invariance model (M1), factorial patterns were set identical in both groups, with factor loadings and intercepts set free across samples; in the metric invariance model (M2) factor loadings were constrained to be equal for each group; in the scalar invariance model (M3) factor loadings and intercepts were identical in both groups. Model fit was calculated through χ^2^ statistical test, the RMSEA and the CFI. Measurement invariance was estimated on the difference between CFIs (ΔCFI). A value of ΔCFI smaller than or equal to |0.010| confirmed the measurement invariance between males and females (Meredith, 1993; Floyd and Widaman, 1995; Little, 1997).

To analyze the nomological network between EI, depression and anxiety, bivariate correlations were computed. To analyze the role of Emotional Intelligence in depression and anxiety, a series of regression analyses were calculated. In the regression model the EIS’ factors were independent variables; dependent variables were anxiety and depression scores. Collinearity was verify through the Condition Index (Barbaranelli and D’Olimpio, 2006).

Finally, to analyze the moderating effect of gender on the association among EI, anxiety and depression, a series of hierarchical regression analyses were performed (Aiken et al., 1991).

SPSS V.16.0 (Spss inc, 2007) was used to calculate descriptive statistics, EFA, correlations, regressions and mediation analysis. AFC, and Invariance Measurement were computed through LISREL V.8.71 (Joreskog and Sorbom, 2004).

## Results

### Descriptive Statistics

Means, standard deviations, normality indices and reliability for each item of the EIS are shown in Table 1. Skewness and kurtosis showed values are in the range ± 1, supporting normal distribution of data (Barbaranelli, 2003). The obtained McDonald’s omega indicated a high internal consistency. Indeed, reliability ranged from 0.854 to 0.860; while the internal consistency of the total scale was 0.860.

**TABLE 1:** Mean, standard deviation, normality indices, and internal consistency of the EIS (*N* = 1725).

Item EIS	Mean	SD	Skewness	Kurtosis	McDonald’s ω if item deleted
1	0.0371	0.968	−1.050	0.800	0.858
2	0.077	0.901	−0.881	0.664	0.856
3	0.039	0.961	−0.814	0.199	0.856
4	0.028	0.960	−0.737	0.138	0.856
5	0.015	0.983	−0.635	−0.256	0.859
6	0.095	0.847	−1.184	0.723	0.860
7	0.015	0.985	−0.490	−0.014	0.859
8	0.100	0.817	−1.436	1.186	0.857
9	0.044	0.968	−0.838	0.135	0.854
10	0.039	0.961	−0.715	−0.101	0.856
11	0.027	0.982	−0.824	0.027	0.856
12	0.028	0.979	−0.286	−0.251	0.855
13	0.020	0.991	−0.270	−0.641	0.858
14	0.046	0.940	−0.875	0.711	0.855
15	0.008	0.996	−0.606	−0.183	0.856
16	0.051	0.934	−0.881	0.778	0.857
17	0.074	0.892	−0.742	0.155	0.856
18	0.037	0.964	−0.626	0.439	0.854
19	0.013	0.982	−0.345	−0.521	0.856
20	0.043	0.960	−0.708	0.294	0.855
21	0.011	0.995	−0.311	−0.653	0.857
22	0.018	0.985	−0.571	−0.177	0.853
23	0.037	0.965	−0.773	0.337	0.855
24	0.061	0.922	−1.030	0.790	0.856
25	0.003	0.990	−0.470	−0.193	0.854
26	0.029	0.989	−0.339	−0.336	0.858
27	0.023	0.977	−0.293	−0.120	0.856
28	0.012	0.988	−0.921	0.113	0.860
29	0.003	0.981	−0.418	−0.119	0.855
30	0.055	0.934	−0.841	0.815	0.854
31	0.043	0.970	−0.781	0.230	0.856
32	0.024	0.979	−0.544	0.044	0.855
33	0.003	0.995	−0.484	−0.287	0.860

### Explorative Factor Analysis

**FIGURE 1:**
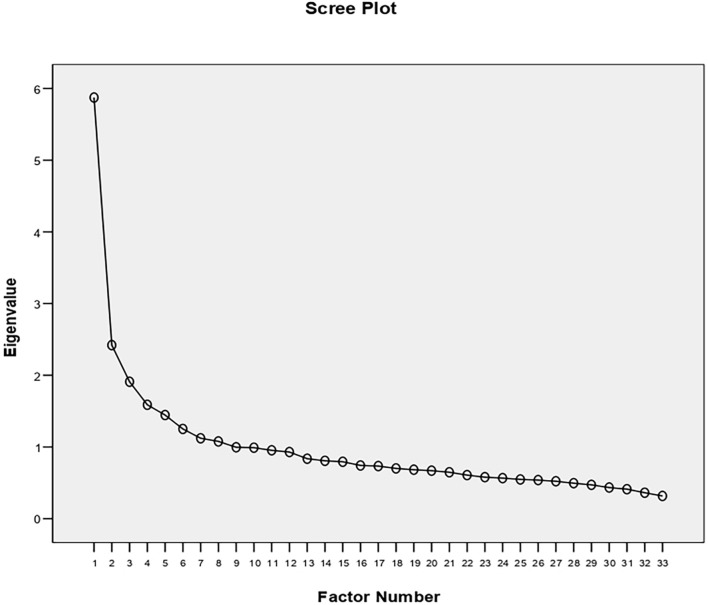
Scree plot of EFA (*N* = 828).

The sub-sample for EFA was composed of 828 participants (males = 302; females = 526), with a mean age of 25.15 (SD = 11.03). The mean age for female’s sample was 23.87 (SD = 9.468); the mean age for male’s sample was 27.39 (SD = 13.055). Bartlett’s Test of Sphericity [χ^2^ (528) = 5914.78; *p* < 0.001] and Kaiser-Meyer-Olkin (KMO = 0.845) showed that data were adequate for factor analysis. Scree plot (Figure 1) and initial eigenvalues (5.873, 2.420, 1.909, 1.588) indicated a four-factor solution. Applying an economic criterion, the four-factor model has been chosen.

Results show that items 1-2-3-6-12-13-26-28-33 can be removed, because of factor loadings < |0.30|, whereas item 12 had double factor loadings on the first and third dimension (0.307 and.326, respectively). The factor model explained the 27.544% of variance Table 2. On the basis of these results, a second EFA without the removed items has been conducted. The second model explained the 32.474% of variance. In this AFE items 10 and 16 had factor loadings < |0.30|. On the basis of these results, a further AFE without the two items has been conducted. This model explained 33.853% of the variance.

**TABLE 2:** Factor loadings, communalities (h^2^) and % of variance explained.

Item EIS	F1	F2	F3	F4	h^2^
20	**0.638**	0.103	0.057	−0.039	0.392
27	**0.529**	0.127	0.015	−0.150	0.301
17	**0.481**	0.051	0.058	0.062	0.269
31	**0.477**	−0.139	0.010	0.140	0.267
7	**0.447**	0.096	0.019	−0.089	0.207
23	**0.441**	0.024	−0.086	0.019	0.238
14	**0.424**	−0.093	−0.088	0.107	0.275
10	**0.374**	−0.198	−0.207	0.135	0.329
16	**0.313**	0.016	−0.023	0.071	0.180
3	0.292	−0.103	−0.197	0.121	0.275
2	0.270	0.074	−0.150	0.089	0.238
28	0.176	−0.170	−0.143	0.099	0.214
13	0.174	0.019	0.004	0.150	0.174
25	0.086	**0.711**	−0.099	−0.065	0.462
18	0.031	**0.602**	−0.122	0.115	0.437
29	−0.064	**0.595**	−0.121	0.199	0.420
5	0.024	**0.459**	0.009	0.026	0.271
32	0.119	**0.395**	−0.079	0.133	0.278
15	0.098	**0.384**	−0.169	−0.063	0.239
26	0.155	0.263	0.109	0.181	0.235
33	−0.043	0.235	0.043	0.213	0.141
22	−0.062	0.091	−**0.835**	0.023	0.530
9	−0.058	−0.013	−**0.640**	0.198	0.442
21	0.041	0.067	−**0.571**	−0.093	0.341
19	0.079	0.225	−**0.484**	−0.089	0.304
12	**0.307**	−0.024	−**0.326**	−0.017	0.314
30	0.016	0.094	−0.006	**0.601**	0.318
4	−0.069	0.147	0.003	**0.574**	0.287
8	0.086	−0.008	−0.045	**0.420**	0.230
24	0.171	0.037	−0.026	**0.364**	0.251
11	0.174	−0.090	−0.112	**0.313**	0.241
1	−0.057	0.086	−0.222	0.247	0.184
6	0.159	0.130	0.088	0.178	0.162
% of variance	15.49	5.00	4.32	2.68	

*N = 828. F1 = Evaluation and Expression of Emotion to Self; F2 = Evaluation and Expression of Emotion to Others; F3 = Social Skills; F4 = Optimism/Mood Regulation. Significant factor loadings are in bold.*

By content analysis of items, the factors have been labeled as follows: “Social Skills”; “Evaluation and Expression of Emotion of Emotion to Others”; “Evaluation and Expression of Emotion to Self; “Optimism/Mood Regulation”.

### Confirmatory Factor Analyses

The sub-sample for CFA was composed of 897 participants (males = 350; females = 547), with a mean age of 26.17 years (DS = 11.60). The mean age for female’s sample was 25.62 (SD = 11.318); the mean age for male’s sample was 27.02 (SD = 11.997). Table 3 shows the goodness-of-fit indices of models tested via EFA. On the basis of results obtained with previous EFA, the items 1-2-3-6-10-12-13-16-26-28-33 were eliminated because of low or double loadings. Without these items the factor model shows better goodness–of-fit indexes.

**TABLE 3:** EIS’s fit indexes.

EIS	χ ^2^	df	RMSEA	90% RMSEA	CFI	GFI	AGFI
Sample*N* = 867	704.19***	203	0.05	0.048;0.057	0.95	0.93	0.92

*df = degrees of freedom; RMSEA = Root-Mean-Square Error of Approximation; CFI = Comparative Fit Index; GFI = Goodness of Fit Index; AGFI = Adjusted Goodness of Fit Index.*

***: p < 0.001.

### Measurement Invariance

Emotional intelligence scale (EIS) scalar invariance was demonstrated across gender. Table 4 shows that ΔCFI values were smaller than | 0.010| for all the model comparisons. In addition, RMSEA showed good values for each model (0.054 for the configural invariance; 0.053 for the metric invariance and 0.053 for the scalar invariance). Therefore, the assumption of equivalent factor loadings and intercepts in males and females was confirmed.

**TABLE 4:** Measurement invariance.

Model	χ ^2^	df	Δ df	RMSEA	RMSEA90%CI	CFI	Δ CFI	Model comparision
Baseline model girls (*n* = 1073)	841.74*; *p* < 0.001	203		0.054	0.0504; 0.0580	0.936		
Baseline model boys (*n* = 652)	568.43*; *p* < 0.001	203		0.053	0.0475; 0.0577	0.951		
M1	1410.18; *p* < 0.001;	406		0.054	0.0505; 0.0566	0.943		
M2	1431.67; *p* < 0.001	424		0.053	0.0495; 0.0555	0.942	−0.001	2 vs 1
M3	1587.84; *p* < 0.001	464		0.053	0.0502; 0.0559	0.935	−0.007	3 vs 2

M1 = model for configural invariance; M2 = model for metric invariance (equal loadings between groups); M3 = model for scalar invariance (equal loadings and intercepts between groups). * = Sig.

### Correlations

Table 5 showed bivariate correlations among the EIS dimensions, depression and anxiety. Results showed that all of the EIS dimensions, in general, had significant and negative correlations with the TDI and the BAI scales in both sexes. In particular, correlations between depression and Emotional Intelligence ranged from −0.412 for “Social Skills” to −0.208 for “Evaluation and Expression of Emotion to Others” in males; correlations ranged from −0.283 for “Social Skills” to −0.025 for “Evaluation and Expression of Emotion to Others” in females. Correlations between anxiety and EIS dimensions ranged from −0.252 for “Social Skills” to −0.001 for “Evaluation and Expression of Emotion to Others” in males, whereas only the “Social Skills” dimension showed a significant and negative correlation with anxiety (*r* = −0.111) in females.

**TABLE 5:** Correlations between EIS dimensions, TDI and BAI, divided for gender.

EIS dimensions	TDI		BAI	
	Males *N* = 335	Females *N* = 704	Males *N* = 288	Females *N* = 407
Social skills	−0.412**	−0.283**	−0.252**	−0.111*
Evaluation and expression of emotion to others	−0.208**	−0.025	−0.001	0.073
Evaluation and expression of emotion to self	−0.293**	−0.265**	−0.004	−0.024
Optimism/Mood regulation	−0.245**	−0.137**	0.022	0.073

*TDI = Teate Depression Inventory; BAI = Beck Anxiety Inventory.*

*p < 0.05. **p < 0.01.

### Regressions of Emotional Intelligence Scale Dimensions on Anxiety and Depression

The Condition Index was < 15 and didn’t show problems of collinearity (Barbaranelli and D’Olimpio, 2006). Anxiety and depression were regressed on EIS dimensions. Table 6 shows the results of regression analyses. The four dimensions of EI explained the 14% of variance of the depression (*F* = 41.904; *p* < 0.001) and the best predictor was the “Social Skills” (β = −0.290, *t* = −9.238; *p* < 0.001). In addition, the four dimensions of EI explained the 5% of variance of anxiety (*F* = 8.832; *p* < 0.001) and the best predictor of anxiety was “Social Skills” (β = −0.216, *t* = −5.260; *p* < 0.001).

**TABLE 6:** Regression analysis of EIS dimensions with anxiety and depression as dependent variables.

	TDI (*N* = 1039)		BAI (*N* = 695)	
Social skills	β	t	β	t
	−0.290	−9.238***	−0.216	−5.260***
Evaluation and expression of emotion to others	0.082	2.590**	0.089	2.185*
Evaluation and expression of emotion to self	−0.198	−6.075***	−0.025	−0.594
Optimism/Mood regulation	0.002	0.046	0.118	2.747**
p	< 0.001		<0.001	
R^2^	0.14		0.05	
AR^2^	0.14		0.04	
F	41.904		8.832	

p = Significance; R^2^ = R Square; AR^2^ = Adjusted R Square. *p < 0.05. **p < 0.01. ***p < 0.001.

### The Moderating Effect of Gender on the Relationship Among Emotional Intelligence, Depression and Anxiety

To study the potential moderating effect of gender on the relationship among EI, depression and anxiety, a series of hierarchical regression analyses were performed. Gender was entered as predictive variable in the first step. In the second step, the total score of EIS was included. In the third step, a multiplicative term EIS x gender was entered. In the fourth step, the total score of the Social Skills as the best predictor of studied illnesses was included. In the last step, a multiplicative term Social Skills x gender was entered. As can be seen from the Table 7, no significant moderating effects of gender on the relationship between the total score of the EI Trait, Social Skills, anxiety and depression was found.

**TABLE 7:** Moderating effect of gender on the relationship between EI, depression and anxiety.

	*β*	*t*	*F*	*R* ^2^	*sr* ^2^
TDI					
Step 1			28.567	0.03	
Gender	–0.164	−5.345***			0.03
Step 2			65.018	0.11	
Gender	–0.197	−6.679***			0.04
EIS	–0.293	−9.939***			8
Step 3			44.404	0.11	
Gender	–0.201	−6.802***			0.04
EIS	–0.257	−7.096***			0.04
EIS × gender	–0.062	–1.713			<0.01
Step 4			74.903	0.13	
Gender	–0.151	−5.180***			0.02
Social Skills	–0.316	−10.863***			0.09
Step 5			51.165	0.13	
Gender	–0.149	−5.119***			0.05
Social Skills	–0.281	−8.138***			0.02
Social Skills × gender	–0.063	–1.83			0
BAI					
Step 1					
Gender					
	–0.117		9.581	0.01	
		−3.095**			0.01
Step 2			5.056	0.01	
Gender	–0.12	−3.157**			0.01
EIS	–0.028	–0.734			<0.01
Step 3			3.722	0.01	
Gender	–0.123	−3.221**			0.01
EIS	0.005	–0.1			<0.01
EIS × gender	–0.051	–1.026			<0.01
Step 4			14.06	0.04	
Gender	–0.116	−3.112**			0.01
Social Skills	–0.159	−4.278***			0.02
Step 5			10.111	0.04	
Gender	–0.115	−3.099*			0.01
Social Skills	–0.116	−2.450**			0
Social Skills × gender	–0.07	–1.471			0

p = Significance; R^2^ = R Square; sr = Part Correlations; *p < 0.05. **p < 0.01. *** p < 0.001.

*0 = female, 1 = male.*

## Discussion and Conclusion

The guidelines of the (National Institute for Clinical Excellence [NICE], 2017) stressed the importance of the prevention of depressive and anxiety disorders. The aim is to increase the emotional competences, such as emotional regulation ability to promote well-being through valid and reliability instruments of assessment of emotional competence and management (Nélis et al., 2011).

According to previous studies, trait EI is associated with depressive and anxiety disorders. The literature further showed that trait EI could be a predictive factor of a better social adaptation (Ciarrochi et al., 2002; Hansenne, 2011). It can be hypothesized that emotional competence and management are linked to a positive mood and a better social support, which offer protection against a wide range of psychopathological conditions. In particular, emotional management moderated the relation between stress and mental health (citation). For this reason, WHO underlined the ability to manage emotions to cope with stressful events during the 2019-nCoV outbreak (World Health Organization [WHO], 2020a).

Despite the role of trait EI in anxiety and depression has been studied, the psychometric properties of the self-report instruments that assess trait EI remain ambiguous. The EIS, whose factorial structure is not clearly defined in literature (Schutte et al., 1998), is among the most used self-report instruments of trait EI.

Considering these problems, the first aim of this study was to analyze the EIS factor structure and its validity in a sample of 1725 participants. Our results showed that the model with four-factor dimensions of trait EI is the best solution. The dimensions were: “Social Skills”; “Evaluation and Expression of Emotion of Emotion to Others”; “Evaluation and Expression of Emotion to Self; “Optimism/Mood Regulation”. These results substantially confirmed previous findings obtained by factor analyses (Petrides and Furnham, 2001; Saklofske et al., 2003; Hussein et al., 2019). Our study evidenced that the “Evaluation and Expression of Emotion” was divided into two different components: evaluation of Self or Others emotions. The “Evaluation and Expression of Emotion to Others” concerns the ability to recognize others’ emotions through the sound of the voice or the facial expression of other people, while the “Evaluation and Expression of Emotion to Self” concerns the ability to recognize and control our own emotions. During childhood, the development of emotional competence allows individuals to know and use their own and others’ emotions to adapt to the socio-cultural context (Zsolnai, 2015). Emotional competence and management comprises three components: the expression of emotions or the ability to communicate positive and negative emotions; the understanding of emotions; the experience of emotions or the ability to recognize emotions (Zsolnai, 2015). The “Emotion Regulation” concerns the monitoring of emotions; while the “Social Skills” concern the tenacity to face all adversities, empathy, and abilities of communication. Finally, our study showed a good level of internal consistency of the four EIS dimensions.

The second aim was to study the measurement invariance of the EIS across gender. Our results confirmed the scalar invariance of the instrument in males and females. These data confirm previous results (Grazzani Gavazzi et al., 2009). Therefore, the four-factor model of EIS is likely unaffected by gender characteristics of participants.

The third aim was to study the association between EIS dimensions, depression and anxiety. Our results showed that high scores in each EIS dimension are associated to lower levels of depression and anxiety in both males and females. These results are confirmed by previous studies (Fernández-Berrocal et al., 2006; Sergi et al., 2012). In addition, our results showed that EIS dimensions are able to predict depression and anxiety scores. In particular, the dimension “Social Skills” showed to be the best predictor of both depression and anxiety scores. Other studies found that “Evaluation and Expression of Emotion to Self” had a predictive role in depression and anxiety (Saklofske et al., 2003; Downey et al., 2010; Sergi et al., 2012; Picconi et al., 2019). Therefore, our results showed that the ability to recognize and to control our own emotions in relation to social context help us to reduce the risk to be affected by depression and anxiety.

Finally, our study showed no moderating role of gender on the relationship between trait EI and depression. These results suggest that the association among EI, anxiety and depression is not gender moderated.

Some limitations of the present study need to be mentioned: EIS measurement invariance was not tested in relation to age; lastly, the association between EI emotional, anxiety and depression should be tested also in a clinical sample composed by people with depression or anxiety disorders. Our study shows that trait EI can play an important role in managing stress and negative emotions also in people with psychological disorders.

In conclusion, the present study provides important new insights into the associations between trait EI, gender, anxiety and depression in a sample of participants without specific mental disorders. EI can help people to manage emotions linked to negative events and to successfully understand emotions in others (Sulaiman, 2013). Indeed, our study showed that “Social Skills” was the best predictor of anxiety and depression. In addition, we found no moderating role of gender in the association among EI, anxiety and depression. This result is confirmed by the EIS measurement invariance for gender. The relationship between EI and psychological disorders can have a positive effect on individual life. Emotional intelligence can improve the quality of peer relations, increase assertiveness and regulate the deleterious effects of attentional bias. Depressed and anxious individuals suffer from attentional bias, in which attentional resources are allocated to identify above all negative events or situations. Indeed, new interventions in psychotherapy and in education context based on creating situations that elicit emotions could be proposed. In particular, these interventions could be focused on “naming emotions,” discerning emotional states and attributing appropriate meaning to moods.” These aspects reduce depression and anxiety. Indeed, poor emotional regulation maximizes the impact of stressful events and it leads to worry and rumination that discern emotions in a dysfunctional way (Salovey et al., 2002). Several studies explain the poor cognitive task performance in depressed individuals as a consequence of hyper-activation in frontal and parietal brain areas. This hyper-activation is related to the difficulty to deactivate limbic regions, which affect the efficiency of cognitive processes reducing the cognitive control on thoughts (Hamann et al., 2004; Harvey et al., 2005; Jones et al., 2010).

The major limitation of the work is the absence of a randomized clinical trial, with a control and an experimental group, that permits a more massive generalization of results. Another limitation is the representativeness of our sample. Indeed, the most of our sample was composed by university students.

## Data Availability Statement

The raw data supporting the conclusions of this article will be made available by the authors, without undue reservation.

## Ethics Statement

The studies involving human participants were reviewed and approved by the Department of Medicine and Aging Sciences, University of Chieti, Italy. The patients/participants provided their written informed consent to participate in this study.

## Author Contributions

MS designed the study, recruited the sample, wrote the manuscript, and collaborated in editing the final manuscript. LP assisted with the design of the study, collaborated in the data analyses, and collaborated in writing the manuscript. MT assisted with the data analyses, collaborated in writing the manuscript, and assisted with the design of the study. ArS assisted with the design of the study and collaborated in editing the final manuscript. SE assisted with the data analyses and revised the article. AnS assisted with the data analyses and collaborated in writing the manuscript. All authors contributed to the article and approved the submitted version.

## Conflict of Interest

The authors declare that the research was conducted in the absence of any commercial or financial relationships that could be construed as a potential conflict of interest.

## Publisher’s Note

All claims expressed in this article are solely those of the authors and do not necessarily represent those of their affiliated organizations, or those of the publisher, the editors and the reviewers. Any product that may be evaluated in this article, or claim that may be made by its manufacturer, is not guaranteed or endorsed by the publisher.
